# Integration of Random Forest Classifiers and Deep Convolutional Neural Networks for Classification and Biomolecular Modeling of Cancer Driver Mutations

**DOI:** 10.3389/fmolb.2019.00044

**Published:** 2019-06-11

**Authors:** Steve Agajanian, Odeyemi Oluyemi, Gennady M. Verkhivker

**Affiliations:** ^1^Graduate Program in Computational and Data Sciences, Schmid College of Science and Technology, Chapman University, Orange, CA, United States; ^2^Department of Biomedical and Pharmaceutical Sciences, Chapman University School of Pharmacy, Irvine, CA, United States

**Keywords:** cancer driver mutations, machine learning classifiers, ensemble-based machine learning features, random forest, deep learning, convolutional neural networks, drug discovery

## Abstract

Development of machine learning solutions for prediction of functional and clinical significance of cancer driver genes and mutations are paramount in modern biomedical research and have gained a significant momentum in a recent decade. In this work, we integrate different machine learning approaches, including tree based methods, random forest and gradient boosted tree (GBT) classifiers along with deep convolutional neural networks (CNN) for prediction of cancer driver mutations in the genomic datasets. The feasibility of CNN in using raw nucleotide sequences for classification of cancer driver mutations was initially explored by employing label encoding, one hot encoding, and embedding to preprocess the DNA information. These classifiers were benchmarked against their tree-based alternatives in order to evaluate the performance on a relative scale. We then integrated DNA-based scores generated by CNN with various categories of conservational, evolutionary and functional features into a generalized random forest classifier. The results of this study have demonstrated that CNN can learn high level features from genomic information that are complementary to the ensemble-based predictors often employed for classification of cancer mutations. By combining deep learning-generated score with only two main ensemble-based functional features, we can achieve a superior performance of various machine learning classifiers. Our findings have also suggested that synergy of nucleotide-based deep learning scores and integrated metrics derived from protein sequence conservation scores can allow for robust classification of cancer driver mutations with a limited number of highly informative features. Machine learning predictions are leveraged in molecular simulations, protein stability, and network-based analysis of cancer mutations in the protein kinase genes to obtain insights about molecular signatures of driver mutations and enhance the interpretability of cancer-specific classification models.

## Introduction

Deep sequencing studies have enabled a detailed characterization of cancer genomes and unveiled important gene-specific signatures of somatic mutations (Davies et al., 2002; Bardelli et al., 2003; Futreal et al., 2004; Samuels et al., 2004; Stephens et al., 2004, 2005; Wang et al., 2004; Sjoblom et al., 2006; Greenman et al., 2007; Wood et al., 2007; Vogelstein et al., 2013; Watson et al., 2013). The steadily growing amount of data generated in cancer genomic studies and next-generation sequencing (NGS) have been the impetus behind formation of international cancer genomic projects and development of large bioinformatics data resources such as Cancer Genome Atlas (TCGA), Genomics Data Commons Portal (https://portal.gdc.cancer.gov/) (Weinstein et al., 2013; Jensen et al., 2017), COSMIC database (http://cancer.sanger.ac.uk) (Forbes et al., 2015), and the International Cancer Genome Consortium (ICGC) (Hudson et al., 2010; Zhang et al., 2011; Klonowska et al., 2016; Hinkson et al., 2017). The Cancer Gene Census of the Catalog of Somatic Mutations in Cancer (COSMIC) database has grown from 291 well-characterized cancer genes (Futreal et al., 2004) to more than 500 entries (Forbes et al., 2015) where some cancer genes can be commonly mutated across cancer types, while other genes are predominantly cancer-specific. The cBio Cancer Genomics Portal (https://www.cbioportal.org/) is an open-access resource for exploration of large cancer genomics data sets (Cerami et al., 2012; Gao et al., 2013). These datasets have allowed for comprehensive genome-wide analyses of genetic alterations in multiple tumor types (Poulos and Wong, 2018). A relatively small fraction of somatic variants known as driver mutations have considerable functional effects and can be acquired over time as a result of a range of mutational processes, rather than inherited (Haber and Settleman, 2007; Lawrence et al., 2013; Vogelstein et al., 2013). A comprehensive analysis of cancer driver genes and mutations has provided classification of 751,876 unique missense mutations, producing a dataset of 3,442 functionally validated driver mutations (Bailey et al., 2018). Another significant dataset of 1,049 experimentally tested and functionally validated driver mutations (Ng et al., 2018) has expanded our knowledge of cancer-causing variants in oncogenes and tumor suppressor genes. TCGA organized the Multi-Center Mutation Calling in Multiple Cancers (MC3) network project which generated a comprehensive and consistent collection of somatic mutation calls for the 10,437 tumor samples dataset (Ellrott et al., 2018). Computational approaches that assess the impact of somatic mutations are often characterized by different basic assumptions, types of input information, models, and prediction targets such as driver gene or driver mutation (Gonzalez-Perez et al., 2013; Cheng et al., 2016).

A number of somatic variant callers based on various statistical and machine learning approaches are now available for somatic mutation detection, including MuTect2 (Cibulskis et al., 2013), MuSE (Fan et al., 2016), VarDict (Lai et al., 2016), VarScan2 (Koboldt et al., 2012), Strelka2 (Kim et al., 2018), SomaticSniper (Larson et al., 2012), and SNooPer (Spinella et al., 2016). A deep convolutional neural network (CNN) approach termed DeepVariant can identify genetic variation in NGS data by discerning statistical relationships around putative variant sites (Poplin et al., 2018). To facilitate systematic and standardized somatic variant refinement from cancer sequencing data, random forest (RF) models and deep learning (DL) approach were utilized, showing that these machine learning techniques could achieve high and similar classification performance across all variant refinement classes (Ainscough et al., 2018). A machine learning approach called Cerebro increased the accuracy of calling validated somatic mutations in tumor samples and outperformed several other somatic mutation detection methods (Wood et al., 2018).

Many computational methods have been proposed for prediction of cancer driver genes. Some of these approaches use cohort-based analysis to detect driver genes, including ActiveDriver (Reimand and Bader, 2013), MutSigCV (Lawrence et al., 2013), MuSiC (Dees et al., 2012), OncodriveCLUST (Tamborero et al., 2013), OncodriveFM (Gonzalez-Perez and Lopez-Bigas, 2012), and OncodriveFML (Mularoni et al., 2016). The success of hybrid methods for scoring coding variants has indicated that integration of different tools may enhance predictive accuracy for both coding and non-coding variants (Li et al., 2015). A deep learning-based method (deepDriver) predicts driver genes by CNN trained with mutation-based feature matrix constructed using similarity networks (Luo et al., 2019). Since many methods are often found to predict distinct or partially overlapping subsets of cancer driver genes, a consensus-based strategy was recently proposed, showing considerable promise and outperforming the individual approaches (Bertrand et al., 2018). A unified machine learning-based evaluation framework for analysis of driver gene predictions compared the performance of these methods, showing that the driver genes predicted by individual tools can vary widely (Tokheim C. et al., 2016; Tokheim C. J. et al., 2016).

Computational methods designed to identify driver mutations have become increasingly important to facilitate an automated assessment of functional and clinical impacts (Gnad et al., 2013; Ding et al., 2014; Martelotto et al., 2014; Raphael et al., 2014; Cheng et al., 2016). Functional computational prediction methods include Sorted Intolerant From Tolerant (SIFT) (Sim et al., 2012), PolyPhen-2 (Adzhubei et al., 2010), Mutation Assessor (Reva et al., 2011), MutationTaster (Schwarz et al., 2010), CONsensus DELeteriousness score of missense mutations (Condel) (Gonzalez-Perez and Lopez-Bigas, 2011), Protein Variation Effect Analyzer (PROVEAN) (Choi et al., 2012), and Functional Analysis Through Hidden Markov Models (FATHMM) (Shihab et al., 2013). Cancer-specific High-throughput Annotation of Somatic Mutations (CHASM) (Carter et al., 2009; Douville et al., 2013; Masica et al., 2017), Cancer Driver Annotation (CanDrA) (Mao et al., 2013), and FATHMM (Shihab et al., 2013). Many new approaches have recently addressed a problem of locating driver mutations within the non-coding genome regions (Piraino and Furney, 2016). The identification of cancer mutation hotspots in protein structures has been a fruitful approach for identifying driver mutations (Dixit et al., 2009; Dixit and Verkhivker, 2011; Gao et al., 2013; Gauthier et al., 2016; Niu et al., 2016; Tokheim C. et al., 2016; Tokheim C. J. et al., 2016). To consolidate functional annotation for SNVs discovered in exome sequencing studies, a database of human non-synonymous SNVs (dbNSFP) was developed (Liu et al., 2011, 2013, 2016; Dong et al., 2015; Wu et al., 2016). This resource allows for computation of a total of 48 functional prediction scores for each SNV, including 32 functional prediction scores by 13 approaches and 15 conservation features (Wu et al., 2016). In our recent investigation, two cancer-specific machine learning classifiers were proposed that utilized 48 functional scores from dbWGFP server in classification of cancer driver mutations (Agajanian et al., 2018).

In this work, we explore and integrate RF and DL/CNN machine learning approaches for prediction and classification of cancer driver mutations. We first explore the ability of CNN models to identify and classify cancer driver mutations directly from raw nucleotide sequence information without relying on specific functional scores. The performance of these classifiers was compared to RF and gradient boosted tree (GBT) methods to provide a comparative analysis of various classification models. These raw sequence-derived scores are advantageous because they can be obtained for any mutation with a known chromosome and position, whereas the functional scoring features can be limited to subsets of genomic mutations. By developing a successful classification scheme that could leverage information from raw DNA sequences, the universe of classifiable mutations can be greatly expanded leading to more general and robust machine learning tools. The results of this study reveal that CNN models can learn high importance features from genomic information that are complementary to the ensemble-based predictor scores traditionally employed in machine learning classification of cancer mutations. We show that integration of the DL-derived predictor score with only several ensemble-based features can recapitulate the results obtained with a large number of functional features and improve performance in capturing driver mutations across a spectrum of machine learning classifiers. Machine learning predictions are leveraged in biophysical simulations and network analysis of protein kinase oncogenes to obtain more detailed functional information about molecular signatures of activating driver mutations, aiding in the interpretability of cancer mutation classifiers.

## Materials and Methods

### Mutational Datasets and Feature Selection

**Figure 1:**
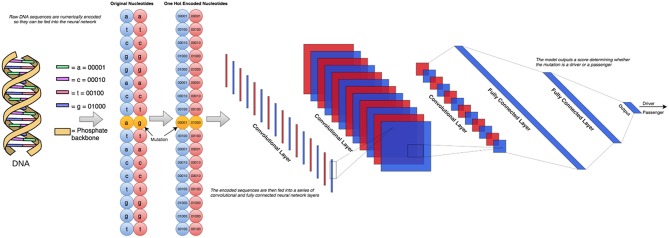
The schematic workflow diagram of the CNN approach employed in this study. To determine the optimal architecture, we performed a grid search over a total of 72 different neural network architectures. These 72 architectures consisted of between 1 and 3 convolutional layers and 1–3 fully connected layers following. The number of nodes in each of these layers was also varied between 2 and 256 in powers of 2. The simplest architecture covered in this search contains 1 convolutional layer with 2 filters feeding into 1 fully connected layer with 2 nodes, and the most complex would have 3 convolutional layers feeding into 3 fully connected layers, all containing 256 nodes.

In our earlier study (Agajanian et al., 2018) we used RF classifier to predict cancer driver mutations using a combination of two golden datasets (Mao et al., 2013; Martelotto et al., 2014). Here, we expanded this dataset by adding the predicted cancer driver mutations and passengers from the analysis of missense mutations in Cbioportal database (Agajanian et al., 2018). By leveraging the earlier analysis, we created a dataset consisting of functionally validated 6,389 cancer driver mutations and 12,941 passenger mutations. The driver/passenger classifications for 2,570 of these mutations were present in the two aforementioned golden datasets, and our RF classifier made predictions on the remaining 16,760 missense mutations from the Cbioportal database. Given the performance level of our model (Agajanian et al., 2018), we conjectured that a combination of the two golden datasets and the missense mutations in the Cbioportal database would yield an informative dataset for the current study. The initially selected features for RF predictions were obtained from dbWGFP web server (Wu et al., 2016) of functional predictions for human whole-genome single nucleotide variants (Supplementary Table S1). A total of 32 sequence-based, evolutionary and functional features identified in our previous study (Agajanian et al., 2018) were initially used for machine learning experiments with the new dataset of cancer mutations. In cancer driver mutation predictions, traditional input data contain distinct features that cannot be directly applied to CNN models due to their lack of spatial meaning. Using the chromosome and the position on that chromosome that corresponded to the mutated nucleotide, we could retrieve the surrounding nucleotides of the mutation of interest to perform classification with only this raw string of nucleotides. To represent the original nucleotide and its mutated version, we placed two nucleotide sequences on top of each other, one containing the original string, and the other contained the mutated version. This would only result in a one nucleotide difference between the two, allowing to effectively utilizing the sliding window format of the CNN models. The schematic workflow diagram of the CNN approach employed in this study is presented in Figure 1.

**Figure 2:**
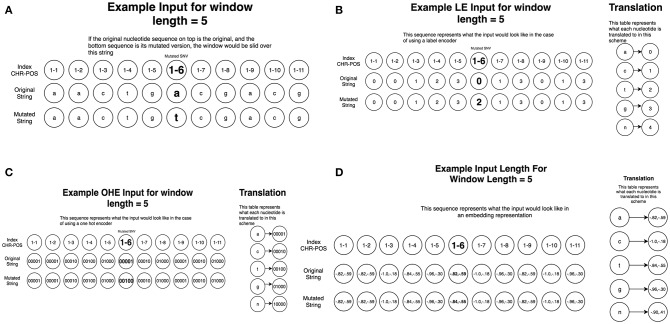
Preprocessing of the nucleotide information for CNN machine learning of cancer driver mutations. Two different preprocessing techniques were then applied to the dataset to allow it to be passed into the CNN model in the numerical form: label encoding and one-hot encoding. **(A)** A schematic diagram of window sliding protocol. To represent the original nucleotide and its mutated version, two nucleotide sequences are placed on top of each other, one containing the original string, and the other contained the mutated version. This representation allows to utilize the sliding window format of the CNN models. **(B)** A schematic diagram of label encoding preprocessing protocol. Label encoding assigns each nucleotide its own unique ID (A->0, C->1 etc.) This imposes an ordering on the nucleotide sequences. **(C)** A schematic diagram of one hot encoding preprocessing protocol. One-hot encoding assigns each nucleotide its own bit encoded string (A -> [0,0,0,0,1], C-> [0,0,0,1,0]). This tends to be a favorable preprocessing function for weight-based classifiers because no artificial ordering is imposed on the samples. **(D)** A schematic diagram of embedding preprocessing scheme created with the word2vec algorithm.

To create this dataset, we parsed information from University of California, Santa Cruz (UCSC) Genome Browser (http://genome.ucsc.edu/) (Tyner et al., 2017) which takes a chromosome (CHR) and a position (POS) on that chromosome as arguments and returns back all nucleotides within the sequence. Using the dataset consisting of 6,389 driver mutations and 12,941 passengers, we created 5 different datasets of various window sizes around each given CHR/POS pair. The explored window sizes (10, 50, 100, 500, and 5,000) produced nucleotide strings of length 21, 101, 201, 1,001, and 10,001, respectively. To represent the type of mutation (A->C, A->G, etc.) we stacked two of the same nucleotide sequences on top of each other, having one contain the original nucleotide at the position passed in initially, and the other containing the mutated version (Figure 2A). This operation resulted in a total input matrix size of (2, 21), (2, 101), (2, 201), (2, 1001), and (2, 10001), respectively. Three different preprocessing techniques were then applied to the dataset to allow it to be passed into the CNN model in the numerical form: label encoding (Figure 2B), one-hot encoding (Figure 2C; Goh et al., 2017), and embedding (Figure 2D). Label encoding involves assigning each nucleotide its own unique ID (A->0, C->1, etc.) This imposes an ordering on the nucleotide sequences that may have implications for the neural network learning (Figure 2B). This technique was implemented using the Scikit-learn LabelEncoder package for the Python programming language. We also tried one-hot encoding the dataset by assigning each nucleotide its own bit encoded string (A -> [0,0,0,0,1], C-> [0,0,0,1,0]) (Figure 2C). This tends to be a favorable preprocessing function for weight-based classifiers because no artificial ordering is imposed on the samples. This technique tends to be the default representation choice for categorical variables due to how it is interpreted. Because each nucleotide gets its own index in a 5 bit string, a 1 in any particular index means that nucleotide is present in that location. For example, since A->[0,0,0,0,1], this can essentially be read as “There are 0 ‘n,' 0 ‘g,' 0 ‘t,' 0 ‘c,' and 1 ‘a' nucleotides present at this location.” Since the one-hot encoding preprocessing technique lengthens the string, the resulting dimensionalities were (2, 105), (2, 505), (2, 1005), (2, 5005), and (2, 50005), respectively. The final preprocessing technique employed for the DNA sequences involved learned embeddings created with the word2vec algorithm (Mikolov et al., 2013). This technique analyzes the sequential context of the nucleotides assigning them a numeric representation in vector space. Using this representation, the nucleotide segments with similar meaning in the word2vec model would yield similar vectors in an N-dimensional representation. This technique was implemented using the Word2Vec model from the genism library for the Python programming language. Since the vocabulary in this application is fairly small, consisting of only 5 bit components, we chose to convert the nucleotide to 2 dimensional vectors which is sufficient to effectively encode this set. This resulted in the input sizes (2, 42), (2, 202), (2, 402), (2, 2002), and (2, 20002), respectively (Figures 1, 2). The implementation and execution of these three preprocessing techniques provides adequate and efficient nucleotide representations for the CNN classifier.

### Machine Learning Models

We used and compared performance of tree based classifiers and DL/CNN machine learning models. For the tree based methods, we used previously established protocol for obtaining hyper-parameters (Agajanian et al., 2018). The model training and tuning was done using Scikit-learn free software machine learning library for the Python programming language (Pedregosa et al., 2011; Biau, 2012). The Keras framework was used for training, validation and testing of CNN models (Erickson et al., 2017). We initially held out 20% of the data in a stratified manner as a testing set so that it had the same distribution of passengers/drivers as the total dataset. We then used the remaining 80% of the dataset as the training set to learn and tune its hyper-parameters. To choose between the hyper-parameters attempted, we test our model out on unseen data so that we have an unbiased estimate of its performance. To do this, we performed 3-fold cross validation, splitting the training set up into three equal sized portions. The model trains on two of them, and makes predictions on the third. This is repeated three times so that each of the three portions has been predicted on. A workflow diagram of the CNN approach (Figure 1) was carefully engineered to determine the optimal architecture. For this, we performed a grid search over a total of 72 different neural network architectures. These 72 architectures consisted of between 1 and 3 convolutional layers and 1–3 fully connected layers following. The number of nodes in each of these layers was also varied between 2 and 256 in powers of 2. The simplest architecture covered in this search contains 1 convolutional layer with 2 filters feeding into 1 fully connected layer with 2 nodes, and the most complex would have 3 convolutional layers feeding into 3 fully connected layers, all containing 256 nodes. The ReLU activation function was used, which returns max (0, X). All 72 different architectures (Table 1) were tested using this cross-validation algorithm and the architecture that had the highest F1 score across all 3-folds was chosen. Our neural networks were trained for 100 epochs, which means that they will pass through the entire dataset 100 times to complete their training. In between each epoch, the model recorded its predictions on the validation fold, and the epoch with the best performance on the validation set was recorded. Dropout was applied in between layers, so that inputs into a layer are randomly set to 0 with a certain probability. This prevents the neural network from overfitting, forcing it to learn without random features present. The best architecture was used for predictions on the test set.

**Table 1:** The parameters of displayed CNN architectures in classification of cancer driver mutations.

**Architecture**	**# Layers**	**# Nodes per layer**
0	2	32,2
1	3	16,8,2
2	3	16,16,2
3	3	32,16,2
4	3	32,8,2
5	3	64,32,2
6	3	64,16,2
7	4	64,64,16,2
8	4	128,64,16,2
9	4	128,64,32,2
10	5	128,64,32,16,2

To assess the performance of each model, Accuracy, Recall, Precision, and F1 score were calculated to measure the performance of classification models. These parameters are defined as follows:

(1) $$\begin{array}{l} Accuracy=\frac{TP+TN}{all};\;Precision=\frac{TP}{TP+FP} \end{array}$$

(2) $$\begin{array}{l} Recall=\frac{TP}{TP+FN};\;F_{1}=2\frac{Precision\;*\;Recall}{Precision+Recall} \end{array}$$

True Positive (TP) and True Negative (TN) are defined as the number of mutations that are classified correctly as driver and passenger mutations, respectively. False Positive (FP) and False Negative (FN) are defined as the number of mutations that are misclassified into the other mutational classes. Precision is defined as the amount of positive samples the model predicts correctly (true positives) divided by the true positives plus the false positives. Recall is defined as true positives divided by true positives plus false negatives. The model performance was evaluated using receiver operating characteristic area under the curve. The receiver operating curve (ROC) is a graph where sensitivity is plotted as a function of 1-specificity. The area under the ROC is denoted AUC. The sensitivity or true positive rate (TPR) is defined as the percentage of non-neutral mutations that are correctly identified as driver mutations:

(3) $$\begin{array}{l} Sensitivity=TPR=\frac{TP}{TP+FN} \end{array}$$

The specificity or true negative rate (TNR) is defined as the percentage of mutations that are correctly identified as passengers:

(4) $$\begin{array}{l} Specificity=TNR=\frac{TN}{TN+FP} \end{array}$$

In combination, these scores allow us to differentiate models by providing evaluation options to properly asses a model's performance. We relied on the F1 score, precision and recall as the primary discriminatory measures that can assess the quality of classification more reliably than accuracy. Under this data distribution, a model that only predicted passenger would yield an accuracy of 66.95%, but an F1 score of 0. In the case that two model's exhibited the same F1 score, we used the AUC measure to break the tie. The AUC measure is derived from the fact that the output of these classification models is a likelihood value between 0 and 1. A powerful classifier learns a likelihood function that consistently maps instances of the negative class to likelihoods lower than the positive class. A model that is reliable able to do this would receive an AUC of 1, whereas a model that only predicted the negative class would also receive an AUC of 0.

### Bimolecular Simulations of Cancer Mutation Effects: Rigidity Decomposition and Protein Stability Analysis

We used FIRST (Floppy Inclusion and Rigid Substructure Topography) approach (Jacobs et al., 2001; Rader et al., 2002; Chubynsky and Thorpe, 2007) and the Python-based Constraint Network Analysis (CNA) interface (Hespenheide et al., 2002; Kruger et al., 2013; Pfleger et al., 2013a,b) to analyze partition of rigid and flexible regions in a set of protein kinases with the predicted cancer driver mutations. The employed parameters are consistent with our previous studies of protein kinases (Stetz et al., 2017). Protein stability computations that evaluated the effect of cancer driver mutations on the functional forms of the ErbB kinases were performed using CUPSAT (Cologne University Protein Stability Analysis Tool) (Parthiban et al., 2006, 2007). This approach was successfully adopted for the energetic analysis of cancer mutation hotspots (Dixit et al., 2009; Dixit and Verkhivker, 2011). We also employed the Foldx method (Guerois et al., 2002; Schymkowitz et al., 2005; Tokuriki et al., 2007; Van Durme et al., 2011) that allows for robust assessment of mutational effects on protein stability. These calculations were done with the user interface for the FoldX force field calculations (Schymkowitz et al., 2005) implemented as a plugin for the YASARA molecular graphics suite (Van Durme et al., 2011).

### Protein Structure Network Analysis

For network-based analysis, a graph-based representation of protein structures is employed in which residues are treated as network nodes and inter-residue edges represent residue interactions (Sethi et al., 2009; Vijayabaskar and Vishveshwara, 2010; Stetz and Verkhivker, 2017). NAPS approach (Chakrabarty and Parekh, 2016) was used for construction of the residue interaction networks and subsequent residue-based network centrality analysis. For our analysis, an interaction strength-based graph representation of protein structures was used in which a residue is considered as node in the network and an edge is constructed if the interaction strength between two residues is more than the threshold of 4%. The pair of residues with the interaction *I*_*ij*_ greater than a user-defined cut-off (*I*_min_) are connected by edges and produce a protein structure network graph for a given interaction cutoff *I*_min_. The interaction strength *I*_*ij*_ is considered as edge weight. The edges in the residue interaction networks were weighted based on the defined interaction strength and dynamic residue correlations couplings (Sethi et al., 2009; Stetz and Verkhivker, 2017). Using the constructed protein structure networks, the residue-based betweenness parameters were also computed with the NAPS server (Chakrabarty and Parekh, 2016). The betweenness of residue *i* is defined to be the sum of the fraction of shortest paths between all pairs of residues that pass through residue *i*:

(5) $$\begin{array}{l} C_{b}\left(n_{i}\right)=\overset{N}{\underset{j<k}{\sum }}\frac{g_{jk}\left(i\right)}{g_{jk}} \end{array}$$

*g*_*jk*_ denotes the number of shortest geodesics paths connecting *j* and *k*, and *g*_*jk*_(*i*) is the number of shortest paths between residues *j* and *k* passing through the node *n*_*i*_. Residues with high occurrence in the shortest paths connecting all residue pairs have a higher betweenness values. For each node *n*, the betweenness value is normalized by the number of node pairs excluding *n* given as (*N*−1)(*N*−2)/2, where *N* is the total number of nodes in the connected component that node *n* belongs to.

## Results

### Deep Learning Classification of Cancer Driver Mutations From Nucleotide Information

We began with an attempt to recapitulate our predictions by using various DL/CNN architectures informed by raw nucleotide sequence data evaluated the ability to make predictions based solely on raw genomic information. The inclusion of the three different preprocessing techniques allowed us to select the most informative representation of the nucleotides. The one hot encoded sequences yielded the model with the best performance, and for clarity of presentation we report only the dimensions and performance of the one hot encoded model. This preprocessing model resulted in input matrices of size (2, 105), (2, 505), (2, 1005), (2, 5005), and (2, 50005) corresponding to the different window sizes (10, 50, 100, 500, 1,000) surrounding the original nucleotide. It is worth noting that the embedding algorithm also learned meaningful representations of the nucleotides. The missing place indicator, “n,” was predictably separated from the original nucleotides, which were arranged in 2 neat clusters (Figure 2D). Cluster 1 consisted of the adenine and tyrosine nucleotides, and cluster 2 consisted of the guanine and cytosine nucleotides. These two clusters are easily identified due to the fact that their constituent components are very close to each other while simultaneously being far away from the other cluster.

**Figure 3:**
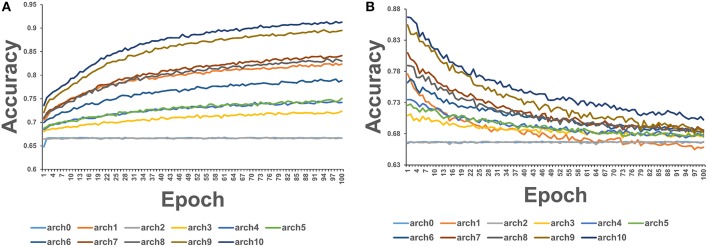
The average accuracy of CNN model using exclusively nucleotide information. **(A)** Average accuracy across all 3-folds on an epoch by epoch basis on the training set with the sliding window size = 10. **(B)** Average accuracy across all 3-folds on an epoch by epoch basis on the validation set with the sliding window size = 10.

We employed 72 different DL architectures (Table 1) and the results for the window size of 10 are presented since they revealed more variance (Figure 3). The figures below display the 10 best performing models out of the 72 attempted. The training accuracy continued to increase for the duration of training (Figure 3A), while on the validation testing set of cancer mutations, the best DL/CNN architecture achieved an average validation accuracy of 86.68% with an F1 score of 0.61 (Figure 3B). Interestingly, we found that the DL model seemed to learn early on, overfitting with each successive epoch (Figure 3B). In fact, the model achieved its highest validation accuracy on the first epoch, and proceeds to decline as learning proceeds in subsequent epochs. Furthermore, the AUC score of the model as well as the F1 score consistently stayed the same throughout all of the process. This is further contextualized by the tree based method's performance on the same dataset. The GBT classifier exhibited an F1 score of 0.57 with an average validation accuracy of 66.59%, and the RF classifier exhibited an F1 score of 0.58 and an average validation accuracy of 69.86%. We analyzed predictions by the DL/CNN model by assigning the predicted values for the entire dataset as a separate new feature termed DL score. Although we probed a variety of different architectures and several nucleotide-encoding protocols, a direct brute-force application of DL/CNN models to predict driver mutations only as a function of surrounding nucleotides appeared to be challenging. As a result, we suggested that a diverse set of more informative features may be required to recapitulate the level of robust performance achieved in our earlier work with sequence-based conservation and functional features (Agajanian et al., 2018).

We first used the RF classifier on the cancer mutation dataset with functional and conservation features obtained from dbWGFP server and adopted in our previous study (Agajanian et al., 2018). A database of human non-synonymous SNVs (dbNSFP) was developed as a one-stop resource for analysis of disease-causing mutations (Liu et al., 2011, 2013, 2016; Dong et al., 2015; Wu et al., 2016) storing 8.58 billion possible human whole-genome SNVs, with capabilities to compute a total of 48 functional prediction scores for each SNV, including 32 functional prediction scores by 13 approaches, 15 conservation features from 4 different tools including ensemble-based predictors RadialSVM, LR, and MSRV scores. The initially selected features were obtained from dbWGFP web server of functional predictions for human whole-genome single nucleotide variants that provided 32 functional prediction scores and 15 evolutionary features (Agajanian et al., 2018). Functional prediction scores refer to scores that predict the likelihood of a given SNV to cause a deleterious functional change in the protein, and evolutionary scores refer to scores providing different conservation measures of a given nucleotide site across multiple species (Supplementary Table S1). Some of the score features (SIFT, PolyPhen, LRT, Mutation Assessor, MutationTaster, FATHMM, RadialSVM, LR, MSRV, and SinBaD) can be applied only to SNVs in the protein coding regions, while other scores (Gerp++, SiPhy, PhyloP, Grantham, CADD, and GWAVA) can evaluate SNVs spreading over the whole genome (Supplementary Table S1). The ensemble-based scores RadialSVM and LR are integrated features that used machine learning approaches to combine information from 10 individual component scores (SIFT, PolyPhen-2 HDIV, PolyPhen-2 HVAR, Gerp++, MutationTaster, Mutation Assessor, FATHMM, LRT, SiPhy, PhyloP) (Agajanian et al., 2018).

**Figure 4:**
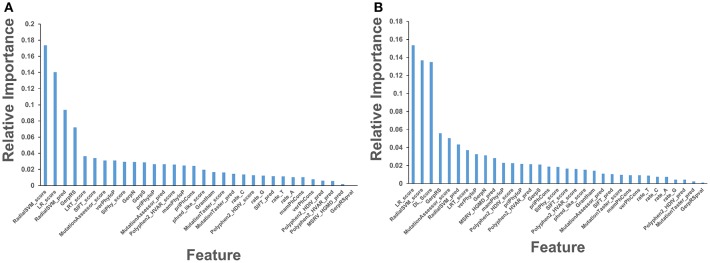
Feature importance of the RF machine learning model on the cancer mutation dataset. The dataset consists of functionally validated 6,389 cancer driver mutations and 12,941 passenger mutations. The initially selected features for RF predictions were obtained from dbWGFP web server (Wu et al., 2016) of functional predictions for human whole-genome single nucleotide variants (Supplementary Table S1). The test set contained 20% of the samples from the original dataset, ensuring that the distribution of drivers and passengers was equivalent to that of the original dataset. The training set was subjected to recursive feature elimination process, resulting in a final dataset of 32 features. **(A)** Feature importance of 32 functional and sequence conservation features with DL score feature produced by CNN model excluded. **(B)** Feature importance of 33 features with the DL score included in the RF classification. The feature importance values are shown in blue filled bars and annotated. Feature importance is measured using the information value and weight of evidence criteria.

In this baseline experiment we evaluated feature performance of 32 input features on the expanded dataset (Figure 4A). Similar to our previous investigation (Agajanian et al., 2018), we found that the ensemble-based scores LR and RadialSVM considerably overshadowed the contributions of other features (Figure 4). By adding DL score to the original 32 features, we applied the RF model for predicting cancer driver mutations with this expanded set of features. The first question was to analyze feature importance of the RF model with the DL score included and determine whether the nucleotide-based scoring feature can contribute to the prediction performance in a meaningful and appreciable way (Figure 4). In the second round of RF classification experiments, we added DL score to the original list of 32 features (Figure 4B). Strikingly, the DL score ranked third following the ensemble-based LR and RadialSVM scores (Figure 4B). Moreover, it was evident that these three feature scores completely dominated feature importance distribution, with the DL score contributing almost as much as the ensemble-based RadialSVM feature (Figure 4B). Quite remarkably, the DL-based score derived by CNN exclusively from primary nucleotide information can deliver significant information content and enrich predictions.

**Figure 5:**
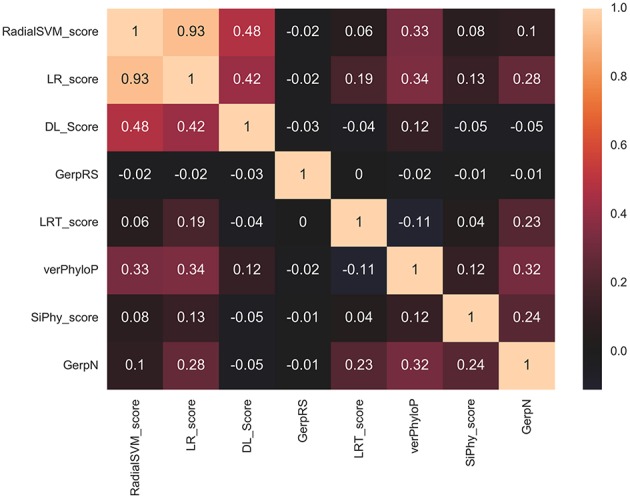
The pairwise Spearman's rank correlation heat map between different prediction scores. The heat map of pairwise Spearman's rank correlation coefficients is shown for top 8 ranking features in the RF classification of cancer mutations with a total of 33 features with DL score included. The high ranking features include ensemble-based RadialSVM, LR scores along with DL score produced by CNN model solely from the raw nucleotide information.

Using Spearman's rank correlation coefficient, we computed the pairwise correlations between different prediction scores (Figure 5). In this analysis, we found that the two dominant feature scores RadialSVM and LR are only moderately correlated with DL score, with the correlation coefficient of 0.486 and 0.423, respectively. Interestingly, RadialSVM and LR scores are more significantly correlated, suggesting that these ensemble-based features could be complementary with the nucleotide-based DL score. Accordingly, we argued that a combination of these dominant and yet complementary scores may allow for feature reduction and more robust performance of the RF classification models.

### Integration of CNN Predictions With Ensemble-Based Features in Classification Models of Cancer Driver Mutations

**Figure 6:**
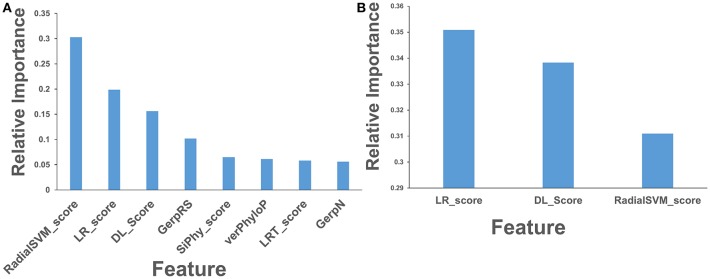
Feature importance of the RF model on the cancer mutation dataset with the reduced number of features. **(A)** Feature importance ranking based on RF classification with only 8 most informative features. **(B)** Feature importance ranking based on RF classification with only 3 top features that included ensemble-based RadialSVM, LR scores, and DL score produced by CNN model. The feature importance values are shown in blue filled bars and annotated. Feature importance is measured using the information value and weight of evidence criteria.

Based on these findings, we evaluated feature selection again aiming to recreate the same accuracy with only 8 features: RadialSVM score, LR score, DL score, GerpRS, LRT score, verPhyloP, SiPhy score, GerpN (Figure 6A). The RF model with only 8 features produced a similar ranking in which the ensemble-based scores and DL score contributed the most (Figure 6A). Other contributing features included evolutionary conservation scores derived from multiple sequence alignments and reflecting functional specificity, such as GerpRS (Davydov et al., 2010), SiPhy (Garber et al., 2009), and PhyloP (Garber et al., 2009) also showed appreciable information score values (Figure 6A). We then tested the performance of the RF model and feature importance by performing machine learning of cancer driver mutations using only 3 top features (Figure 6B).

**Figure 7:**
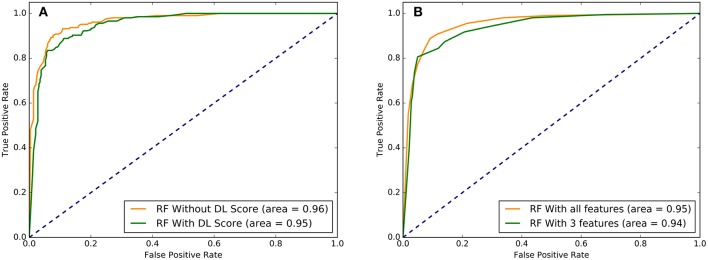
The ROC plots of sensitivity (TPR) as a function of 1-specificity, where specificity (TNR). **(A)** The ROC curves for overall performance of the RF model with 32 functional features excluding DL score (in green) and 33 features that included DL score (in red). **(B)** The ROC curves for the RF model with all 33 features (in green) and with the top 3 performing features that included LR score, Radial_SVM score, and DL score (in red). Higher AUC score indicates better performance. These plots illustrated a comparative performance of machine learning models for top prediction scores.

The predictive performance of the RF models with different set of features was examined using area under the curve (AUC) plots (Figure 7). First, we examined difference in the AUC curves for RF-based classification with 32 functional features and with additional DL score (Figure 7A). The results showed a very similar high-level prediction performance with AUC = 0.95–0.96. It is worth noting that due to high AUC value for RF classification with 32 informative functional features, the addition of DL could not significantly enhance it. However, we showed that this nucleotide-derived predictor score provides an additional information content and is complementary to the ensemble-based RadialSVM score and LR score. In this context, it was instructive to observe that addition of DL score may marginally improve separation between TPR and FPR at higher values of these parameters (Figure 7A).

Strikingly, RF learning model that relied on only 3 top features (RadialSVM score, LR score, and DL score) yielded AUC = 0.94, thereby showing that these features may be sufficient to achieve robust classification of cancer driver mutations on a fairly large dataset of somatic mutations employed in this study. Combined with the findings that DL score only weakly correlated with the ensemble-based scores, we concluded that unexpectedly few highly informative parameters can achieve high level of performance (Figure 7). We then tested several machine learning models including RF, GBTs and support vector machine (SVM) on the dataset with the top 8 features to benchmark performance against the original RF model with 32 features (Agajanian et al., 2018). The performance of classification models was carefully assessed (Table 2). All methods achieved a high classification accuracy of ~90%. The sensitivity values were higher for the SVM and RF models, but all methods yielded similar high performance classification on the dataset with only limited number of major features that included DL score (Table 2).

**Table 2:** The relative performance metrics and statistics of various machine learning models in classification of cancer driver mutations with the top 8 features.

	**Boosted trees**	**SVM**	**Random forest**
Accuracy	0.896	0.890	0.896
F1 score	0.900	0.890	0.900
Precision	0.900	0.890	0.900
Recall	0.900	0.890	0.900
True positive rate	0.850	0.949	0.857
False positive rate	0.112	0.797	0.123
True negative rate	0.115	0.016	0.107
False negative rate	0.913	0.748	0.907

To summarize, our results supported the notion that machine learning-derived ensemble functional predictors may play a central role in classification of cancer driver mutations. The central finding of these machine learning experiments was that combination of ensemble-based features and DL score derived by CNN model from nucleotide information are complementary and when combined can yield classification accuracy comparable and often exceeding the one obtained with a full set of features. The important lesson from this analysis is that integrated high-level features derived by machine learning approaches from primary nucleotide and protein sequence information may be sufficient to predict an important functional phenotype. Although structure-derived features and other functional scores contribute to feature importance ranking and tightly linked with the mutational phenotype, the success of machine learning tools in deciphering predictive features from primary sequence information is encouraging and should be further explored in other applications.

### Leveraging Machine Learning Predictions in Structure-Functional Analysis of Molecular Signatures of Driver Mutations in Oncogenic Protein Kinases

Machine learning driver/passenger classifications typically consider activating, inactivating and inhibitory (or resistant) mutations as drivers, often leaving aside a more detailed characterization and assignment of driver positions. Direct predictions of these specific classes may not be adequately suited for machine learning tools due to smaller datasets. To expand our predictions and aim at extracting a more granular functional information about driver mutations, we conducted rigidity decomposition simulations and analyzed conformational flexibility of the predicted driver positions in protein kinase genes. The objective of this analysis was to facilitate functional validation and interpretation of machine learning results through coarse-grained biophysical simulations as an effective post-processing tool of machine learning classification. In fact, the proposed simulation analysis of mobility at the driver positions allows to expand classification of driver mutations further and characterize activating drivers. Previous studies have suggested that conformational mobility of many oncogenic kinases may be linked with preferential localization of activating cancer mutations in flexible functional regions (Paladino et al., 2015; Kiel et al., 2016; Stetz et al., 2017).

**Figure 8:**
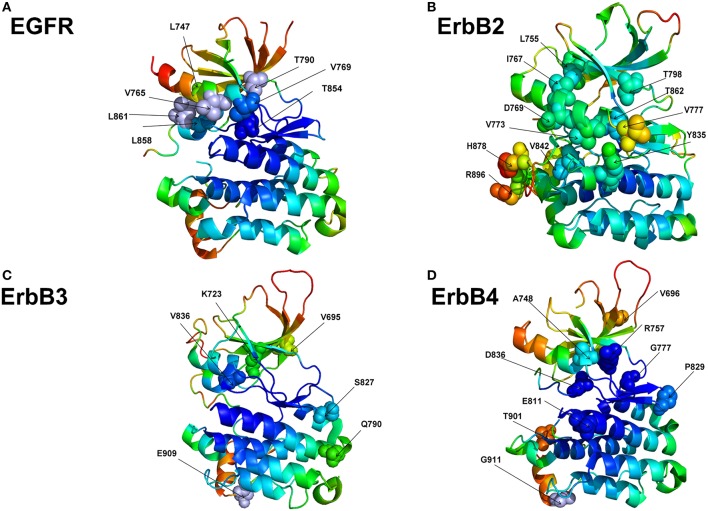
Structural maps of rigidity decomposition and mobility signatures of cancer mutation drivers in the ErbB protein kinases. Structural mapping of rigidity and flexibility regions in the crystal structure of EGFR (pdb id 1XKK) **(A)**, crystal structure of ErbB2 kinase (pdb id 3PP0) **(B)**, crystal structure of ErbB3 kinase (pdb id 3KEX) **(C)**, and crystal structure of ErbB4 kinase (pdb id 3BBT) **(D)**. Crystallographic conformations are colored using a color range from red (highest flexibility) to blue (highest rigidity). The positions of predicted in machine learning cancer driver mutations are shown in spheres (colored according to their mobility level) and annotated.

We examined flexibility of specific functional regions targeted by driver mutations in oncogenic protein kinases and probed functional propensity of these drivers to promote transitions to constitutively active states. The primary focus of this analysis is on the family of the ErbB protein tyrosine kinases (Lemmon and Schlessinger, 2010; Roskoski, 2014). A number of human cancers are associated with mutations causing the increased expression of the ErbB kinases. A large number of activating and drug resistance EGFR mutations have been extensively studied at the molecular and functional levels (Paez et al., 2004; Kobayashi et al., 2005; Zhou et al., 2009; Eck and Yun, 2010). Oncogenic kinase mutants are known to act by destabilizing the inactive dormant kinase form while promoting conformational transitions and stabilization of a constitutively active kinase state—a salient functional characteristic linked with the initiation or progression of cancer (Carey et al., 2006; Wang et al., 2011). We used the crystal structures of the EGFR, ErbB2, ErbB3, and ErbB4 kinases that constitute this family to perform rigidity decomposition and then align the positions of the predicted cancer driver mutations with the structural mobility maps (Figure 8). We examined how the predicted driver mutations for ErbB protein kinases are distributed on the rigidity/flexibility map of the catalytic core and whether the dynamic preferences of mutational sites can be linked with their primary function as activating drivers. To explore these questions, we examined the predicted cancer driver mutations for the ErbB kinase family. Structural mapping of these cancer mutations onto the crystallographic ErbB conformations showed that activating driver mutations are preferentially localized in the flexible regions and target positions where they can readily promote conformational changes to the active form without severely compromising thermodynamic stability (Figure 8).

**Figure 9:**
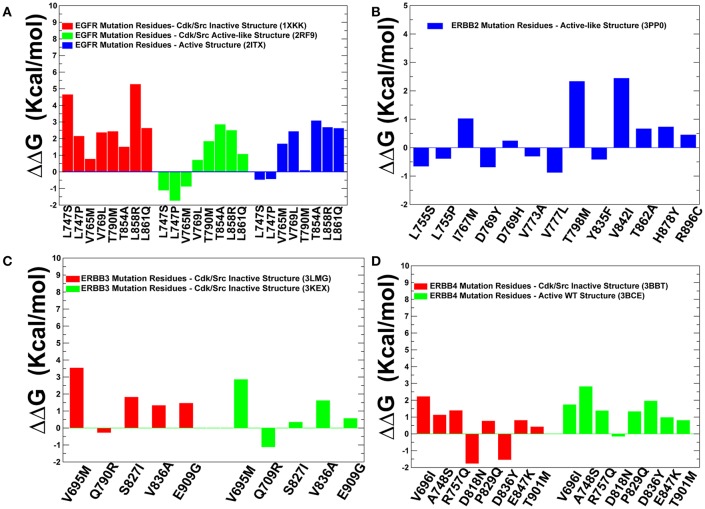
Protein stability analysis of the predicted cancer driver mutations. Protein stability differences calculated between the wild-type and mutants for predicted cancer driver mutations in the ErbB kinases using FOLDx approach. Protein stability changes induced by cancer driver mutations in the inactive and active states of EGFR kinase **(A)**, ErbB2 kinase **(B)**, ErbB3 kinase **(C)**, and ErbB4 kinase **(D)**. Positive values of protein stability changes correspond to destabilizing mutations.

To quantify these arguments further, we also characterized the free energy differences between wild-type and cancer-driver mutations for the ErbB proteins in both inactive and active kinase forms (Figure 9). Since both CUPSAT and FoldX approaches yielded similar results, we illustrated our findings by presenting FoldX-derived protein stability changes (Figure 9). The results of this simulation-driven functional classification of predicted driver mutations were compared with the biochemical and mutagenesis data. The analysis of driver mutations in EGFR confirmed that L858 and L861 positions target flexible regions as can be manifested by classical activating driver mutations L858R and L861Q (Littlefield and Jura, 2013; Red Brewer et al., 2013). The energetics of these activating drivers is consistent with a common mechanism of the constitutive activation of kinases by driver mutations (Figure 9A). This mechanism reflects a combined effect of activating mutations producing a more significant destabilization of the inactive state as compared to the active state, triggering shift of the thermodynamic equilibrium toward the active conformation. We found that some EGFR mutations such as T854A are mapped onto more stable regions of the kinase (Figure 8A) and showed similar destabilization in the inactive and active forms. Accordingly, this predicted cancer driver mutation is likely not activating but rather may be attributed to inhibitory or resistant mutations. Indeed, the recent experimental studies showed that T854A mutation is the acquired mutation causing resistance to known drugs (Bean et al., 2008). Another EGFR mutation V769M/L showed an intermediate level of mobility (Figure 8A) and greater stabilization of the active state. These results are in line with recent functional experiments showing that EGFR-V769M mutation is indeed activating that may explain the role of this driver mutation in the development of multiple lung cancers in a pool of lung cancer patients (Deng et al., 2018).

The positions of almost all predicted driver mutations in ErbB2 kinase target highly flexible regions and can be assigned in our model to activating driver mutations (Figures 8B, 9B). Our previous biophysical simulations and network analysis of activation mechanisms in the ErbB proteins similarly indicated that almost all oncogenic ErbB2 variants are localized in the mobile αC-β4 loop and highly dynamic in their inactive states promoting transition to the active form and causing an uncontrollable activity (James and Verkhivker, 2014). These findings are consistent with the experimental studies (Fan et al., 2008; Aertgeerts et al., 2011). While the majority of somatic mutations in the EGFR and ErbB2 kinases increase the kinase activity, a number of the classified ErbB4 cancer mutants have been shown to inhibit or reduce the kinase activity (Tvorogov et al., 2009). In particular, some cancer-associated mutations of ErbB4 can promote loss of ErbB4 kinase activity as these alterations weaken the important functional interactions in the catalytic core and may interfere with the protein stability. According to experimental data, some cancer mutations have only minor or no effect on kinase activity (V696I, E785K, A748S, P757Q, P829Q, and T901M), while K726R abolishes kinase activity and D818N and D836Q are known as kinase-dead mutations (Tvorogov et al., 2009). We found that predicted cancer driver mutations are mapped onto more stable regions in ErbB4, owing to the greater rigidity of this catalytic domain (Figures 8D, 9D). Accordingly, the respective driver mutations cannot function as activating but rather may cause significant distortions of the kinase structure, causing abolishment of kinase activity which is the functional signature of most cancer drivers in ErbB4 kinase. The performed simulation-driven post-processing of machine learning predictions facilitated *in silico* functional characterization of cancer mutations and allowed to properly assign activating or inhibiting phenotypic effects to a pool of pathogenic kinase variants.

To provide more quantitative insights, we used the predicted cancer mutations in the ErbB kinases and conducted protein structure network analysis to identify whether positions of deleterious mutations would overlap with the global mediating nodes in the interaction networks. The betweenness of a residue node is defined as the number of shortest paths that can go through that node, thus estimating the contribution of the node to the global communication flow in the system. High betweenness nodes can influence the spread of information through the network by facilitating, hindering, or altering the communication between others. According to our hypothesis, cancer mutations may preferentially target the essential mediating residues with a high centrality that play an important role in activity and signaling of protein kinase genes.

**Figure 10:**
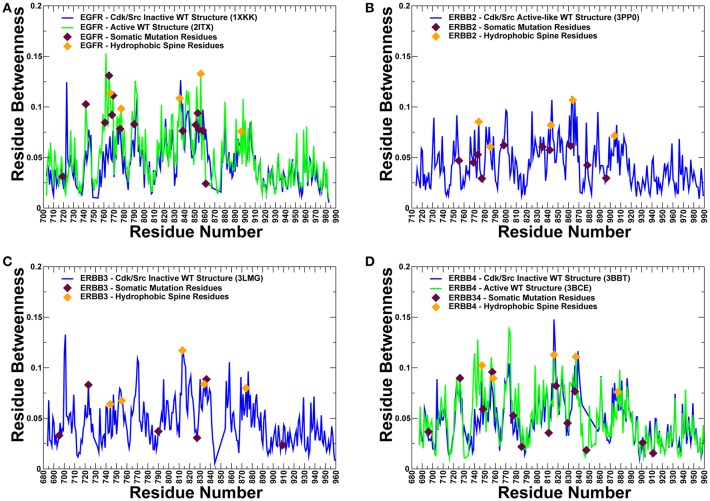
The residue-based betweenness profiles of the ErbB kinase structures. The residue betweenness (residue centrality) profiles for the inactive and active crystal structure states of EGFR **(A)**, ErbB2 **(B)**, ErbB3 **(C)**, and ErbB4 kinases **(D)**. For ErbB2 and ErbB3 only crystal structures of the inactive-type states were available for the analysis. The positions of somatic mutations predicted by machine learning experiments are shown in maroon-colored filled triangles, and residue positions corresponding to the hydrophobic spine residues are shown in orange-colored filled triangles. Protein kinase activation is controlled by two networks of mostly hydrophobic residues that form a regulatory spine (R-spine) and a catalytic spine (C-spine). The EGFR R-spine residues include L777 from the β4-strand, M766 from the C-terminal end of the αC-helix, F856 of the DFG motif in the activation segment, H835 of the HRD motif of the catalytic loop, and D896 of the αF-helix.). The R-spine residues in ErbB2 are M774, L785, F864, H843, and D904. The R-spine residues in ErbB3 are I744, L755, F843, H813, and D874. The R-spine residues in ErbB4 are M747, L758, H816, F837, and D877.

The centrality analysis revealed important differences in the distribution of mediating centers in the ErbB kinase structures (Figure 10). We particularly observed that the betweenness of the active form of EGFR (Figure 10A) and ErbB4 (Figure 10D) was on average higher than for the inactive states. Importantly, the location of the properly classified EGFR mutations with the highest oncogenic potential (L858R, T790M, L838V, V742A, V851A, I853T) corresponds to some of the high centrality peaks of the profile (Figure 10A). In addition, these residues showed appreciable differences in the betweenness values between the inactive to the active states, as the residue centrality in these positions typically increased in the functional active form (Figures 10A,D). These findings suggested that a number of key activating mutations in the ErbB kinases target mediating sites of global allosteric communication in the protein structures. We believe that by adding this significant additional component to our study, we have been able to further quantify and explain the protein rigidity/flexibility analysis of predicted cancer mutations in the kinase genes. In our view, by complementing machine learning predictions with the structural and network-based analyses we can obtain useful insights into mechanisms underlying effects of cancer mutations and also identify limitations of classification models and ways to improve interpretability and trustability of machine learning model approaches.

## Discussion

As large-scale biological data are available from high-throughput assays, and methods for learning the thousands of network parameters have matured, we can now assess feasibility and practicality of using specialized neural network architectures as classification tools for recognizing cancer-causing variants and associated cancer types. Given rapid proliferation and increasing popularity of deep learning tools to address various biological problems, there are several fundamental questions arising in the context of classification of cancer driver mutations. Will deep learning make all other models obsolete? Can deep learning models achieve robust classification and recognition of cancer driver mutations based solely on nucleotide information? What is the role of many functional and structural predictors derived from biophysical perspective in this context? In this work, we have explored and integrated different machine learning approaches for prediction and classification of cancer driver mutations. We first explored the ability of CNN models to identify and classify cancer driver mutations directly from raw nucleotide sequence information without relying on specific functional scores.

The results of this study have demonstrated that while CNN models can learn high level features from genomic information that has sufficiently high importance, accurate classification of cancer mutation driver phenotype using exclusively nucleotide data continues to be challenging. This problem is admittedly more complex than the experimental design suggests, due to the complex nature of protein interactions in the human body. This experimental setup considered only the primary sequence form of the nucleotides, which could only ever partially explain the onset of cancer. The secondary, tertiary, and quaternary form of these same strings would certainly contain more information, due to the folding processes that occur in these steps. Additionally, this technique ignores all of the possible interactions that can be had with other structures in the body, which further dilutes the informational value present in the dataset. As such it's unreasonable to assume that our solely primary sequence based dataset would be able to explain all of the variance present in a complex problem like determining a single mutation's level of effect on the onset of cancer. The experimental inclusion of the different window sizes was also an attempt to allow increasing numbers of surrounding nucleotides to have an influence on our chosen mutation's effect. An obvious assumption here is that more nucleotides would in fact bring in more information. This, however, proved not to hold up as the only dataset that provided any significant variance in performance was the window size = 10 dataset. This suggests that more nucleotides only confuse the model and disallow it from learning informative patterns. This problem could possibly be combatted in future research by testing out larger architectures.

The benefits of integrating CNN-derived predictors obtained from nucleotide information with protein sequence features, evolutionary and functional scores were then carefully examined. By exploring various encoding techniques and an array of different CNN architectures, we have found that neural networks can quickly learn an important functional signal, but can rarely steadily improve the initial performance spike with the number of additional epochs. The juxtaposition of monotonically increasing training accuracy with monotonically decreasing validation accuracy is a telltale sign of overfitting. This suggests that there is only a small amount of useful information that can be learned very early on, and subsequent epochs only cause the model to learn noisy patterns that are only exhibited in the training set. It is difficult to determine exactly what was learned by the model due to the black box nature of neural networks, however due to the short path to optimality it is safe to say that any learned concepts cannot be overly complex. We have pursued a synergistic strategy in which the prediction score generated by CNN models was integrated with physics-based functional, structural and evolutionary conservation features. The important lesson of this analysis was the revelation that CNN-derived features may be complementary to the ensemble-based predictors often employed for classification of cancer mutations. These other scores are not calculated from raw sequence based techniques, which supports this DL score as a novel inclusion into a portfolio of scores due to its unique derivation.

By combining deep learning-generated score with only two main ensemble-based functional features, we were able to achieve a high performance level for cancer driver mutations. The robustness of this approach was verified by several traditional machine learning classifiers, including RF, SVM, and GBTs. We have found that integration of CNN-derived predictor score with only several ensemble-based features can recapitulate the results obtained with a large number of functional features and improve performance in capturing driver mutations across a spectrum of machine learning classifiers. Our findings have also demonstrated that synergy of nucleotide-based deep learning scores and integrated metrics derived from protein sequence conservation scores can allow for robust classification of cancer driver mutations with a reduced number of highly informative features. This is an interesting and highly informative result, as the law of parsimony holds for machine learning models so simpler models with comparable performance are typically preferred over their more complex counterparts. Part of this model complexity includes the number of features that a model relies on. As such a reduction in features is a universally positive outcome. In addition to the improved quality of the model, it also expands the universe of predictable nucleotides that are available to us since we depend only on the presence of two ensemble-based scores. The DL score can be derived for any mutation with known coordinates so this is not a limiting factor. In this respect our initial goal of expanding the nucleotides we can make predictions for was partially achieved. This increase in the generalization of these models facilitates the logical conclusion of driver classification efforts, accurately classifying all known nucleotides.

While machine learning approaches can often produce robust and accurate predictors, the ultimate goal of research is fundamental understanding of the underlying phenomena which requires a mechanistic model of the world. In this context, machine learning predictions are leveraged in biomolecular simulations to enable analysis of cancer mutation mechanisms and obtain a more specific information about an important subset of cancer mutations, activating drivers. The results of our investigation suggested that through integration of machine learning classification and biomolecular simulations of cancer mutations we can often validate the predictions and facilitate a more detailed functional analysis of activating driver mutations. These findings can provide insight and new angle to the problem of interpretability of “black box” machine learning results. By carefully inspecting predictions of machine learning models in the context of dynamic and energetic signatures of mutational sites for oncogenic protein kinases, this study offered instructive strategy for simulation-based post-processing of machine learning predictions and detailed functional specification of cancer driver mutations. The proposed synergistic integration of machine learning and biomolecular simulations into a single computational platform allows to rapidly process large datasets and make robust predictions on functionally significant cancer drivers. The results of this study may also inform and guide design of targeted and personalized therapeutic agents combating a spectrum of mutational changes occurring in cancer.

## Data Availability

Publicly available datasets were analyzed in this study. This data can be found here: https://www.cbioportal.org/.

## Author Contributions

GV and SA conceived and designed the research. SA and OO performed the research. SA, OO, and GV analyzed the results and wrote the manuscript. GV wrote the final version of the manuscript and supervised the project.

### Conflict of Interest Statement

The authors declare that the research was conducted in the absence of any commercial or financial relationships that could be construed as a potential conflict of interest.
